# The influence of circulating anti-Müllerian hormone on ovarian responsiveness to ovulation induction with gonadotrophins in women with polycystic ovarian syndrome: a pilot study

**DOI:** 10.1186/1477-7827-11-115

**Published:** 2013-12-17

**Authors:** Saad A Amer, Ahmad Mahran, Ayman Abdelmaged, Ahmad R El-Adawy, Moustafa K Eissa, Robert W Shaw

**Affiliations:** 1 Department of Obstetrics and Gynaecology, Division of Medical Sciences and Graduate Entry Medicine, University of Nottingham, Royal Derby Hospital, Derby DE22 3DT, UK; 2 Faculty of Medicine, Minia University, Minia, Egypt

**Keywords:** Anti-Müllerian hormone, Follicle stimulating hormone, Ovulation induction, Polycystic ovarian syndrome

## Abstract

**Background:**

Women with polycystic ovarian syndrome (PCOS) are known to have elevated circulating Anti-Müllerian hormone (AMH), which has been found to desensitize ovarian follicles to follicle stimulating hormone (FSH). The purpose of this study was to investigate the impact of high circulating AMH on ovarian responsiveness to ovulation induction with gonadotrophins in PCOS women.

**Methods:**

This prospective observational pilot study was conducted in two collaborating Fertility Centres in the UK and Egypt. The study included 20 consecutive anovulatory women with PCOS who underwent 34 cycles of human menopausal gonadotrophin (hMG) ovarian stimulation using chronic low-dose step up protocol. Blood samples were collected for the measurement of serum AMH concentrations in the early follicular (day 2-3) phase in all cycles of hMG treatment. The serum levels of AMH were compared between cycles with good *vs.* poor response. The good response rates and the total dose and duration of hMG treatment were compared between cycles with high *vs.* low serum AMH concentrations.

**Results:**

Cycles with poor response (no or delayed ovulation requiring >20 days of hMG treatment) had significantly (p = .007) higher median{range} serum AMH concentration (6.5{3.2-13.4}ng/ml) compared to that (4.0{2.2-10.2}ng/ml) of cycles with good response (ovulation within 20 days of hMG treatment). ROC curve showed AMH to be a useful predictor of poor response to hMG stimulation (AUC, 0.772; P = 0.007). Using a cut-off level of 4.7 ng/ml, AMH had a sensitivity of 100% and specificity of 58% in predicting poor response. The good response rate was significantly (p < .001) greater in cycles with lower AMH (<4.7 ng/ml) compared to that in those with AMH > = 4.7 ng/ml (100% *vs.* 35%, respectively). All cycles with markedly raised serum AMH levels (> 10.2 ng/ml) were associated with poor response. Cycles with high AMH (> = 4.7 ng/ml) required significantly (p < .001) greater amounts (median {range}, 1087{450-1650}IU) and longer duration (20 {12-30}days) of hMG stimulation than cycles with lower AMH (525 {225-900}IU and 8{6-14}days).

**Conclusions:**

PCOS women with markedly raised circulating AMH seem to be resistant to hMG ovulation induction and may require a higher starting dose.

## Background

Anti-Müllerian hormone (AMH) is a dimeric glycoprotein, which is secreted exclusively by granulosa cells of primary, preantral and small antral follicles (4-6 mm). Its secretion gradually diminishes in the subsequent stages of follicle development and is practically undetectable in follicles larger than 8 mm [1]. AMH has been shown to lower the sensitivity of follicles to circulating follicle stimulating hormone (FSH) [2,3]. Serum AMH concentrations have been correlated with the number of small follicles and hence ovarian reserve. In polycystic ovarian syndrome (PCOS) serum AMH concentration shows a two- to three-fold increase, which corresponds to the two- to three-fold increase in the number of small (2 – 5 mm) follicles [4,5]. This increase in AMH has been implicated in the pathogenesis of PCOS. It has been hypothesised that the high serum AMH levels in PCOS lowers follicular sensitivity to circulating FSH thus preventing follicle selection resulting in follicle arrest at the small antral phase with failure of dominance. AMH also inhibits aromatase activity resulting in reduction of follicle production of oestradiol (E2) [6]. The resulting low levels of E2 may also contribute to the failure of follicle selection. It is therefore possible to hypothesise that high serum AMH levels could adversely affect ovarian responsiveness to gonadotrophin ovulation induction in women with PCOS.

Serum AMH has recently been widely accepted as an excellent predictor of ovarian responsiveness to gonadotrophin treatment in ovulation induction as well as in in-vitro fertilization (IVF) programmes. In women without PCOS, serum AMH has been found to correlate positively with ovarian responsiveness to gonadotrophin stimulation [7,8]. In women with PCOS, there has been no study on the predictive value of circulating AMH during gonadotrophin ovulation induction. Lie Fong and co-workers investigated the changes in circulating AMH, but not its predictive value, in PCOS women receiving gonadotrophin ovulation induction [9]. Although, the title of that paper indicates that AMH is not a useful predictor of ovarian response to gonadotrophin treatment in PCOS women, the study did not investigated that issue at all. Concerning IVF in PCOS women, data on the influence of circulating AMH on the outcomes are conflicting. Xi and co-workers reported negative correlation between serum AMH concentrations and fertilization and pregnancy rates in PCOS women [10]. On the other hand, other studies reported positive correlation between circulating AMH and IVF outcomes including pregnancy rates [11] and number and maturity of retrieved oocytes [12]. Parco and co-workers reported on the diagnostic, but not the predictive, value of circulating AMH during IVF [13]. Guzman and co-workers investigated the predictive usefulness of circulating AMH in PCOS women undergoing in-vitro maturation (IVM) treatment [14].

In two recent studies involving women with PCOS, we have found excessive circulating AMH to be associated with poor ovarian response to laparoscopic ovarian diathermy [15] and clomiphene citrate ovulation induction [16]. To the best of our knowledge, the impact of circulating AMH on gonadotrophin ovulation induction in women with anovulatory PCOS has never previously been investigated. The aim of this study was to assess the predictive value of circulating AMH in PCOS women undergoing ovarian stimulation with gonadotrophins.

## Methods

This prospective observational pilot study was conducted at two collaborating sites including the Derby Fertility Unit, University of Nottingham, UK and the Assisted Conception Unit, Minia University, Egypt. The study included 20 clomiphene citrate-resistant women with PCOS who received 34 cycles of ovulation induction with human menopausal gonadotrophin (hMG) with timed intercourse (TI) or intrauterine insemination (IUI) between November 2009 and March 2011. The inclusion criteria were: age 18 - 39 years, BMI < = 35 kg/m(2), anovulatory infertility and a diagnosis of PCOS based on Rotterdam consensus criteria (two of three criteria: Oligo/anovulation, hyperandrogenaemia and sonographic appearance of polycystic ovaries) [17]. Anovulation was diagnosed when the cycle length was longer than 6 weeks or when mid-luteal serum progesterone concentration was < 10 pmol/L (in women with shorter cycles). Hyperandrogenism was diagnosed either clinically (acne/hirsutism) and/or biochemically (testosterone > 2.5 nmol/l or free androgen index [FAI] >=5). The ovary was considered polycystic on ultrasound scan if it contained >= 12 follicles (2-9 mm in diameter) and/or measured >10 ml in volume. In addition, all participants had proven patency of at least one fallopian tube and normal semen analysis of their male partners according to the 1999 WHO criteria [18]. We excluded women with other causes of anovulation such as thyroid dysfunction and hyperprolactinaemia. Patients with marked hyperandrogenaemia were screened for congenital adrenal hyperplasia (by measuring serum concentration of 17alpha hydroxyl-progesterone) and Cushing syndrome (by measuring urinary free cortisol).

### Ethical approval

Ethics approval for this study was given by the Derby Ethics Committee, UK (REC reference: 09/H0401/60, date 07/10/2009) and by Minia University Hospital Ethics Committee (Egypt). All participants provided informed written consent.

### Outcome measures

The primary study outcome measure was good response to hMG therapy defined as occurrence of ovulation within 20 days of treatment. The secondary study outcome measures included ovulation, pregnancy and cancellation rates and total dose and duration of hMG therapy.

### Gonadotrophin therapy

The chronic low-dose step-up hMG stimulation regimen was utilized in all the cases. One of the investigators (AM) established and standardized the treatment procedures in the two collaborating sites. Human menopausal gonadotrophin (hMG) (Menopur, Ferring, UK) was given starting on cycle day three in a dose of 75 IU alternate days. The aim of treatment was to achieve mono-ovulation. Monitoring of treatment was achieved by serial transvaginal ultrasound scanning and serum oestradiol measurements every other day starting from cycle day nine. Size and number of follicles and serum oestradiol levels were recorded in patients follow up sheets. The dose of hMG was reviewed around stimulation day 10 and if follicular development was unsatisfactory, the dose was increased to 75 IU daily. Further increases of the dose (by adding 75 IU alternate days) were considered at weekly intervals when no satisfactory response was achieved. If a good response was not achieved after 28 days, the cycle was cancelled. A new cycle was commenced with a higher starting dose of Menopur (75 IU per day). When one follicle reached a size of > =18 mm a single dose of 10,000 IU human chorionic gonadotrophin (hCG, Pregnyl, Organon, UK) was given. In patients scheduled for IUI, this was performed 36 hours after the hCG injection.

### Criteria for cycle cancellation

hMG stimulation cycles were cancelled either due to under or over response to treatment. Under response was diagnosed when there was no follicular growth after 14 days of stimulation despite increasing the dose of hMG injections or when follicular growth became arrested after an initial response. Over response was diagnosed when there were three or more follicles ≥ 17 mm and/or E2 levels > 5000 nmol/l.

### Intrauterine insemination

IUI was performed in 12 patients (22 hMG cycles) using fresh semen samples by Specialist Nurses (in the Fertility Unit, Derby, UK) or by the Fertility Clinician (in the Assisted Conception Unit, Minia Egypt). Insemination was carried out using the Rocket DUO 23 cm catheter or Bulp Tip (Embryo Transfer Set) 23 cm in difficult cases (Rocket Medical, USA). Timed intercourse was advised in three cycles, whilst the remaining nine cycles were cancelled as detailed below.

### Luteal phase support

Progesterone support of the luteal phase was commenced on the day of IUI with Utrogestan 200 mg vaginal capsules twice daily (Utrogestan, Ferring, UK) or Prontogest 400 mg vaginal pessaries twice daily (Prontogest, Marcyrl, Egypt).

### Diagnosis of pregnancy

Urine pregnancy tests were performed 15 days after IUI. If the pregnancy test was positive a transvaginal ultrasound scan was arranged after two weeks.

### Blood collection and AMH assays

A venous blood sample was collected on cycle day 2 of all cycles of hMG treatment to measure baseline serum concentrations of AMH. Further blood samples were collected for the measurement of serum AMH concentrations on days 9, 15 and 21 of treatment cycle one. The samples were immediately transferred to the research laboratory of each centre, centrifuged for 15 minutes at 2000 X g at 4°C and stored at -80°C for later analysis for AMH concentrations. Stored samples collected in Minia University were transferred to the research laboratory of the UK site (Derby Medical School) for analysis. Serum samples were assayed for AMH in duplicate using enzyme-linked immunosorbent assay kit (Uscn Life Science Inc., USA), which is a sandwich enzyme immunoassay for the in vitro quantitative measurement of AMH in serum, plasma and other biological fluids. This kit has an intra- and inter-assay coefficient of variation of less than 10% and less than 12%, respectively. The minimum detectable level of human AMH by this kit was typically > 0.046 ng/ml with a detection range of 0.156-10 ng/ml. The assay has high sensitivity and excellent specificity for detection of human AMH with no significant cross-reactivity or interference.

### Statistical analysis

Data were entered into the Statistical Package for Social Sciences (SPSS) version 17. Unpaired Mann-Whitney U test was used to compare serum AMH levels between cycles with good *vs.* poor response to hMG stimulation (as defined above). Receiver – operating characteristic (ROC) curve analysis was used to evaluate the predictive value of AMH. Chi-square test was used to compare good response rates between cycles with high vs. low AMH concentrations. The total dose and duration of hMG administration were compared between cycles with high *vs.* low serum AMH concentrations using Mann-Whitney U test. A P value < 0.05 was considered statistically significant. Logistic regression analysis was used to determine the independent effect of AMH on ovarian responsiveness to hMG stimulation after adjusting for other confounders including age, BMI, testosterone levels, FAI and ovarian volume. Backward stepwise elimination was used for the multivariate logistic analysis of prediction of patients with good ovarian responsiveness to hMG stimulation. *P* > 0.10 was used as a cut-off level for exclusion of non-significant individual parameters from the prognostic model. The Cox and Snell square measure of goodness of fit was used to check for lack of fit of the final model.

## Results

### Patients’ characteristics

Table 1 shows patients’ demographic, clinical and endocrine characteristics. The baseline median {range} serum concentration of AMH was 5.5 {2.2-13.4} ng/ml, which remained fairly constant throughout the cycle (day 9, 5.7 {2.1-13.2}; day 15, 5.5 {2.3-13.3} and day 21, 5.6 {2.1-13.1} ng/ml.

**Table 1:** Demographic, clinical and baseline endocrine features of 20 anovulatory women with PCOS who underwent hMG ovarian stimulation

**Characteristic**		**Overall (n = 20)**		**Good responders (n = 12)**		**Poor responders (n = 8)**	
		**Mean (SD)**					
*Age (years)*		29.1	(4.2)	28.3	(4.9)	30.3	(2.6)
*Body mass index (kg/m*^*2*^*)*		26.8	(5.3)	27.2	(5.6)	26.3	(5.0)
*Duration of infertility (years)*		4.5	(2.7)	5.0	(3.0)	3.4	(2.8)
*Serum LH (IU/L)*		9.1	(4.3)	9.5	(3.9)	8.5	(5.0)
*Serum FSH (IU/L)*		4.9	(2.4)	5.2	(3.1)	4.5	(0.4)
*Serum LH/FSH ratio*		2.3	(1.4)	2.4	(1.7)	1.9	(1.2)
*Serum testosterone (nmol/l)*		2.3	(0.7)	2.3	(0.6)	2.2	(0.7)
*Free androgen index*		6.4	(2.4)	6.5	(2.4)	6.2	(2.3)
*Ovarian volume (ml)*		11.8	(2.7)	12.0	(3.0)	12.7	(2.4)
		**n (%)**					
***Menstrual cycle***	Regular	2	(10)	2	(17)	0	(0)
	Oligomenorrhoea	12	(60)	8	(66)	4	(50)
	Amenorrhoea	6	(30)	2	(17)	4	(50)
***Hirsutism***	Yes	9	(45)	6	(50)	5	(63)
	No	11	(55)	6	(50)	3	(37)
***Acne***	Yes	11	(55)	6	(50)	3	(37)
	No	9	(45)	6	(50)	5	(63)
***Infertility***	Primary	16	(80)	10	83	6	75
	Secondary	4	(20)	2	17	2	25
***Ethnicity***	Caucasian	8	(40)	4	33	4	(50)
	Mediterranean	11	(55)	7	58	4	(50)
	Asian	1	(5)	1	9	0	(0)

Results are compared between good *vs.* poor responders.

Numerical values are presented as mean (SD) and categorical data are given as n (%). No statistically significant differences were found between good and poor responders.

### Outcome of hMG ovarian stimulation

#### Ovarian responsiveness

Of the 34 cycles of hMG stimulation included in this study, 19 (56%) resulted in a good response (defined as ovulation within 20 days of hMG stimulation). The remaining 15 cycles were considered poor response (defined as either lack of ovulation (n = 6) or ovulation after prolonged (>20 days) hMG stimulation (n = 9). One (5%) of the 19 cycles with good response was associated with three follicles > = 17 mm and was therefore cancelled due to over response.

#### Ovulation and pregnancy rates per cycle

Ovulation occurred in 28 (82%) of the 34 cycles and pregnancy was achieved in three (9%) cycles. Nine cycles (26%) were cancelled either due to lack of response to hMG (n = 8, 23%) or due to over response (three follicles of >17 mm diameter) (n = 1, 3%).

#### Ovulation and pregnancy rates per patient

Amongst the 20 PCOS women participating in this study 17 (85%) ovulated and three (15%) conceived.

### Cycles with good response *vs.* cycles with poor response to hMG stimulation

Base line serum concentrations of AMH, luteinizing hormone (LH), FSH, testosterone, FAI and ovarian volume were compared between cycles with good and cycles with poor response (Table 2). AMH was significantly (p = .007) higher in cycles with poor response compared to cycles with good response.

**Table 2:** Day 2-3 serum hormonal concentrations and ovarian volume in 34 cycles of hMG ovarian stimulation in 20 anovulatory women with PCOS

	Cycles with poor response to FSH stimulation* (n = 15)	Cycles with good response to FSH stimulation** (n = 19)	**p**
*LH (IU/L)*	6.7 (1.3-17.4)	8.6 (5.2-18.9)	.348
*FSH (IU/L)*	4.5 (4.0-6.0)	4.5 (1.0-14.1)	.650
*Testosterone (nmol/l)*	2.6 (0.9-3.6)	2.1 (1.6-3.6)	.705
*FAI*	7.0 (2.0-13.0)	6.0 (3.0-13.0)	.983
*Ovarian volume (ml)*	14.1 (8.5-16.7)	10.9 (8.0-16.7)	.188
*AMH (ng/ml)*	6.5 (5.1-13.4)	4.0 (2.2-10.2)	*.007*

The results are compared between cycles with poor response and those with good response. Values are presented as median (range). Mann-Whitney test was used for comparisons.

*Poor response, is defined as absence of or delayed ovulation (>20 days of **hMG** stimulation).

**Good response is defined as occurrence of ovulation within 20 days of **hMG** stimulation.

### Responders *vs.* non-responders

The number of pregnancies (n = 3) and the number of patients not achieving ovulation (n = 3) were too small to allow meaningful statistical comparisons. The median baseline AMH concentrations in pregnant and non-pregnant women were 3.2 (3.2-13.4) ng/ml and 5.7 (2.2-12.5) ng/ml respectively. The median serum AMH concentration in women achieving ovulation was 5.1 (2.2-13.4) ng/ml and that of patients who did not ovulate was 5.7 (5.3-12.3) ng/ml.

### ROC curve

**Figure 1:**
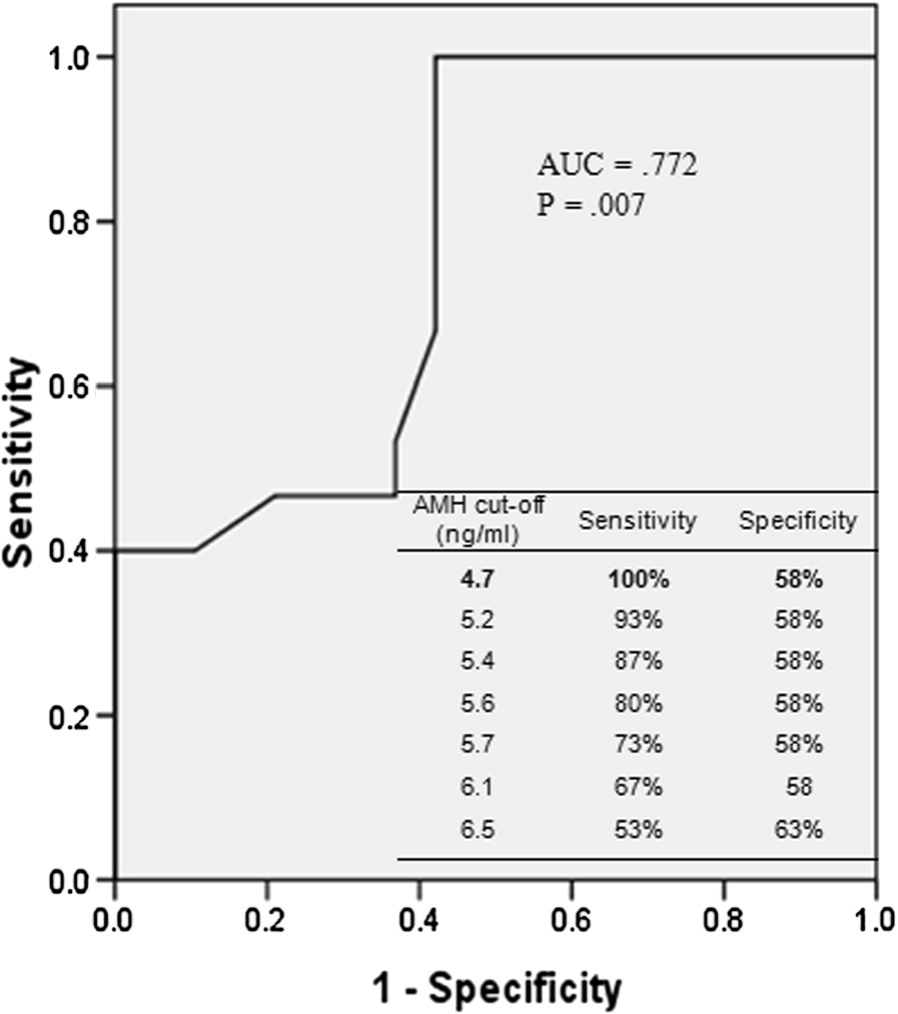
ROC curve of AMH for predicting good response in 34 cycles of hMG ovarian stimulation and possible cut-off values with corresponding specificity and sensitivity in predicting poor response to hMG stimulation.

Using a ROC curve, AMH was found to be a useful predictor of poor response to hMG ovarian stimulation with an AUC of .772 (p = .007) (Figure 1). Different cut-offs of AMH levels in predicting response to hMG stimulation with the corresponding sensitivity and specificity are also shown (Figure 1). Using a cut-off value of 4.7 ng/ml, AMH had a sensitivity of 100% and specificity of 58% in predicting poor response to hMG ovarian stimulation.

### Ovarian response in cycles with high vs*. low AMH*

Using a cut-off AMH value of 4.7 ng/ml (as determined by the ROC curve), the outcomes of hMG ovarian stimulation were compared between cycles with high AMH vs. low AMH levels. Cycles with high AMH levels had significantly lower rates of good ovarian response and higher rates of cancellation (Table 3). The results also showed a trend towards lower ovulation rates in cycles with high AMH levels, but this did not reach statistical significance (Table 3).

**Table 3:** **Clinical outcomes of hMG ovarian stimulation in cycles with high AMH (> = 4.7 ng/ml) *vs. *** **cycles with low AMH (<4.7 ng/ml)**

**Outcome**	**AMH < 4.7 ng/ml (11 cycles)**	**AMH > = 4.7 ng/ml (23 cycles)**	**P**	**RR (95% CI)**
*Good response*	11 (100%)	8 (35%)	*<.001*	2.88 (1.64-5.03)
*Ovulation*	11 (100%)	17 (74%)	.075	1.35 (1.06-1.72)
*Cancellation*	0 (0%)	9* (39%)	*.01*	1.64 (1.18-2.28)
*Over response*	0 (0%)	1 (4%)	.535	1.15 (0.98-1.35)
*Pregnancy*	2 (18%)	1 (4%)	.239	0.21 (0.02-2.50)

Data are presented as n (%). Chi square was used for comparisons.

*Of the 9 cancellations, 8 were due to poor response and 1 was due to over response.

**Figure 2:**
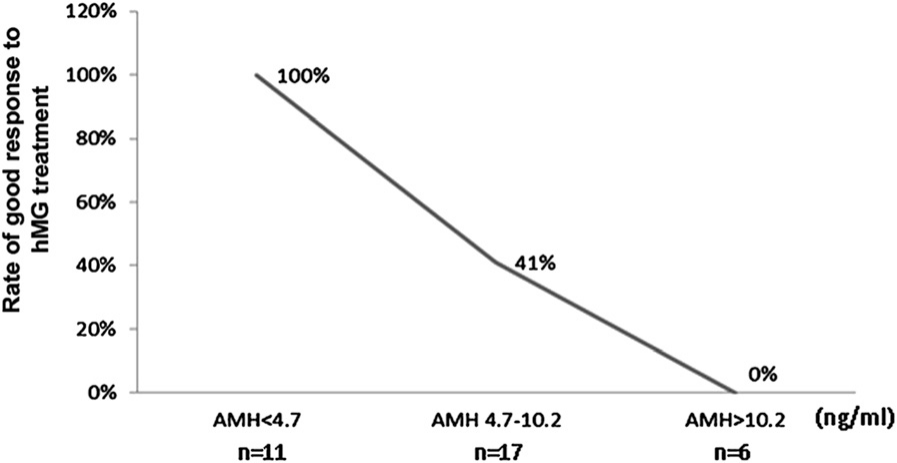
Good response rates in 34 cycles of hMG ovarian stimulation in PCOS women with different serum AMH levels.

Figure 2 illustrates the rates of good response in PCOS women with low (<4.7 ng/ml), moderately elevated (4.7 – 10.2 ng/ml) and markedly elevated (>10.2 ng/ml) serum AMH concentrations. The results show 100% good response rate in women with AMH <4.7 ng/ml and 100% poor response rate in patients with AMH > 10.2 ng/ml.

### AMH and the total dose and duration of hMG stimulation

Spearman's correlation revealed a statistically significant positive correlation between baseline serum AMH concentration and the total dose and duration of hMG administration (Figure 2). No significant correlation was found between AMH and the maximum serum oestradiol concentration (data not shown).

### Total dose and duration of hMG stimulation in cycles with low *v.* high AMH

hMG stimulation cycles with high baseline AMH (≥4.7 ng/ml) required significantly greater amounts and longer duration of hMG stimulation than cycles with lower AMH (Table 4).

**Table 4:** **comparison of the total dose and duration of hMG in cycles with high (≥4.7 ng/ml) *vs. *** **low AMH (<4.7 ng/ml)**

**FSH ovarian stimulation**	**All cycles**	**AMH ≥ 4.7 ng/ml (23 cycles)**	**AMH < 4.7 ng/ml (11 cycles)**	**P**
	**(n = 34)**			
*Total dose (IU)*	788 (225-1650)	1087 (450-1650)	525 (225-900)	<.001
*Duration (days)*	15 (6-30)	20 (12-30)	8 (6-14)	<.001

Data are presented as median (range) and Mann Whitney test was used for comparison.

### Correlation between circulating AMH and demographic and clinical characteristics

**Figure 3:**
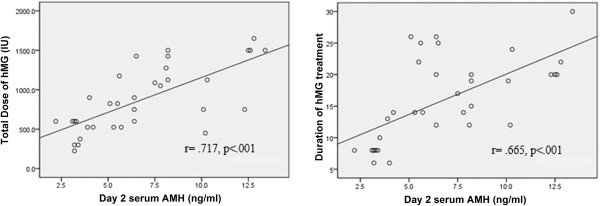
Spearman correlations between baseline AMH and the dose and duration of hMG stimulation in 34 cycles.

**Figure 4:**
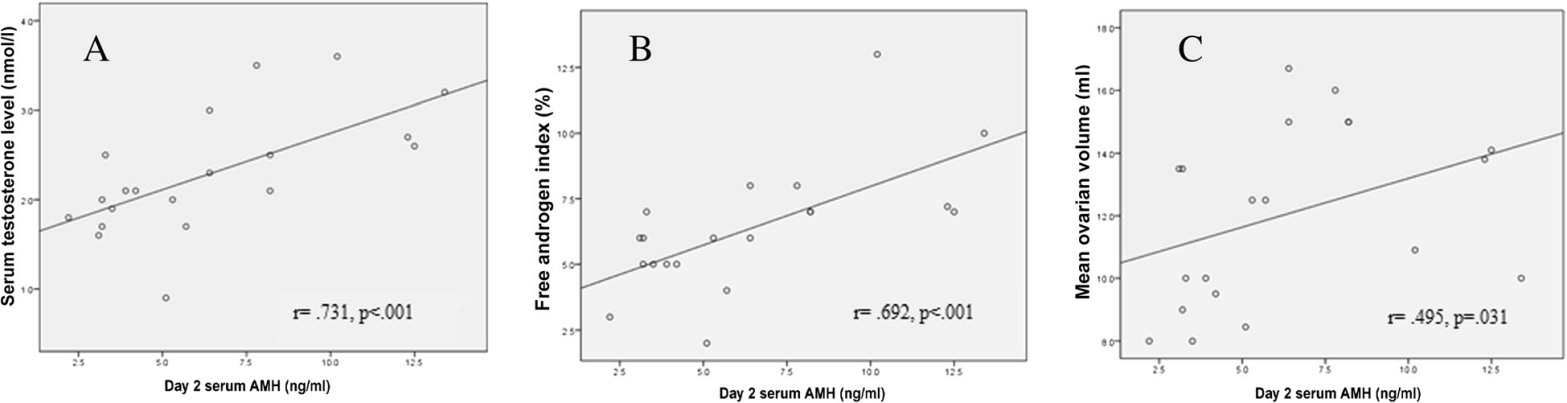
Spearman correlations between baseline serum AMH levels and (A) serum Testosterone, (B) Free androgen index and (C) Ovarian volume in 20 women with PCOS undergoing hMG ovarian stimulation.

Spearman’s correlation revealed a strong positive correlation between baseline AMH levels and serum testosterone levels, FAI and ovarian volume (Figures 3 and 4). No significant correlation was found between AMH and patients’ age, BMI, LH or FSH (data not shown).

### Endocrine features and ovarian volume in PCOS women with high *vs.* low AMH

PCOS women with high AMH had significantly higher serum concentration of testosterone, FAI and ovarian volume as shown on Table 5.

**Table 5:** **gonadotrophins, androgens and ovarian volume in PCOS women with high *vs. *** **low baseline serum AMH concentrations**

	**AMH < 4.7 ng/ml (n = 8)**	**AMH ≥ 4.7 ng/ml (n = 12)**	**P**
*LH (IU/L)*	8.2 (5.2-12.5)	10.0 (1.3-18.9)	.33
*FSH (IU/L)*	4.7 (3.9-14.1)	4.5 (1.0-6.0)	.67
*Testosterone (nmol/l)*	2.0 (1.6-2.5)	2.6 (0.9-3.6)	.04
*FAI*	5.0 (3.0-7.0)	7.0 (2.0-13.0)	.03
*Ovarian volume (ml)*	9.8 (8.0-13.5)	13.9 (8.5-16.7)	.01

### Logistic regression

Logistic regression analysis including AMH, age, BMI, testosterone, FAI and ovarian volume as independent predictors of ovarian responsiveness to hMG stimulation, showed AMH to be the most important independent factor. The final logistic regression model had an *R*(2) (Cox and Snell) of 0.558.

## Discussion

In this study, we have evaluated the impact of circulating AMH on the outcome of ovarian stimulation in 20 women with anovulatory PCOS undergoing 34 cycles of gonadotrophin treatment. To the best of our knowledge, this is the first study to address this important issue in women with PCOS. We found circulating AMH levels to be negatively correlated with ovarian response to hMG. Furthermore, we have identified a cut-off level of serum AMH concentration (4.7 ng/ml), above which the chances of good ovarian response were markedly reduced from 100% (in women with lower AMH) to 35%. Furthermore, we have demonstrated that PCOS women with higher levels of AMH require higher doses of hMG and longer duration of treatment. In addition, we have shown significantly higher cancellation rates in patients with higher AMH.

The negative influence of high AMH levels on ovarian responsiveness to gonadotrophin therapy may reflect the correlation between rising serum AMH levels and increasing severity of PCOS. It is assumed that severe PCOS is associated with an increased number of small antral follicles (the only source of AMH) resulting in excessive AMH secretion. Another possible explanation is the negative effect of excessive AMH secretion on the sensitivity of growing antral follicles to the administered gonadotrophin preventing folliculogenesis [2,3].

These findings suggest that high circulating AMH is associated with ovarian resistance to gonadotrophin ovarian stimulation. However, it is important to note that over response to hMG could still occur in women with moderately elevated circulating AMH (4.7 – 10.2 ng/ml). On the other hand, none of the PCOS patients with markedly raised circulating AMH achieved a good response to hMG treatment.

These findings are consistent with our previous studies on the impact of circulating AMH on the outcome of laparoscopic ovarian drilling and clomiphene citrate [15,16]. It is therefore possible to hypothesise that PCOS women with relatively high serum levels of AMH seem to be resistant to all methods of ovarian stimulation. Further studies are required to confirm this hypothesis and to establish ways of overcoming this resistance. For instance, studies could look into the benefit of adjustment of the doses of CC or gonadotrophin according to the level of circulating AMH.

Our findings seem to contradict the previous study by Lie Fong and co-workers [9] who suggested that serum AMH is not an accurate marker of ovarian response to low dose gonadotrophin ovulation induction in patients with PCOS. However, this assumption was not based on a direct assessment of the predictive value of AMH, but was based on the finding that AMH levels remained stable during the gonadotrophin treatment cycle. It is not clear how this conclusion was reached. Although, circulating AMH remains constant during the treatment cycle, which is consistent with our findings, it varies considerably between different patients. Different serum AMH levels may have different effects on follicular response to gonadotrophin treatment.

Interestingly and in contrast to the above, high serum AMH levels are known to predict over response to gonadotrophin ovarian stimulation in women without PCOS [7,8]. However, the spectrum of circulating AMH is different in women with and without PCOS. In other words, what is considered high AMH level in normal women would be an average level in PCOS women. It is therefore possible to hypothesise that there is an optimum level of serum AMH, which is necessary for successful ovarian stimulation. This level represents the overlap between women with and without PCOS. Levels above and below the optimum AMH values are associated with poor ovarian response to stimulation. This hypothesis requires confirmation by further studies.

It should be noted that our cut-off AMH level applies only to the AMH kit used in this study (Uscan assay). It may, however, be possible to work out the equivalent levels for other kits if the differences between these kits are determined. We have previously reported that the AMH values obtained by Uscan assay are approximately 50% of the values obtained by the IOT and Gen II assays, which are more widely used in clinical practice [16].

Our findings in this study could help in counseling women with PCOS regarding the chance of success and the risks of over response with gonadotrophin therapy. In addition, pre-treatment measurement of serum AMH levels could help in determining the starting dose of hMG. Patients with markedly raised AMH levels can be given a high starting dose of gonadotrophin. However, another study will be required to determine the starting dose of hMG based on the serum level of AMH. In addition to saving time and money, this approach may also reduce patients’ frustration from failure of several-month treatment before reaching the effective dose.

### Strengths and limitations

The main strength of this study is its prospective design with inclusion of consecutive patients fulfilling the study inclusion criteria. The main limitation of this study is the relatively small number of patients included. However, serum AMH levels are known to be generally stable with minimal variation allowing small studies to show significant differences. Furthermore, the findings in this study are supported by multiple lines of statistical evidence. We have used several statistical tests, which have all consistently showed the same effect of circulating AMH on ovarian response to hMG treatment. Another limitation is the possible variation in study procedures in the two centres involved. However, the procedures were standardized in the two centres before starting the study. One of the investigators (AM) was involved with establishing the procedures in the two centres.

## Conclusions

In conclusion, PCOS women with markedly elevated serum AMH levels seem to be resistant to gonadotrophin ovarian stimulation and may require higher doses of this treatment. Pre-treatment measurement of serum AMH concentrations may therefore be a valuable predictor of success and may help in determining the starting dose. We therefore recommend that all PCOS women requiring gonadotrophin therapy would benefit from measuring their baseline circulating AMH.

## Abbreviations

AMH: Anti-Müllerian hormone; E2: Oestradiol; FAI: Free androgen index; FSH: Follicle stimulating hormone; hCG: Human chorionic gonadotrophin; hMG: Human menopausal gonadotrophin; IUI: Intrauterine insemination; IVF: In-vitro fertilisation; IVM: In-vitro maturation; LH: Luteinizing hormone; PCOS: Polycystic ovarian syndrome; ROC: Receiver operating characteristic; TI: Timed intercourse.

## Competing interests

The authors declare that they have no competing interests.

## Authors’ contributions

SA conceived the idea, designed the study, carried out statistical analysis and prepared the manuscript. AM prepared the protocol, obtained ethics approval, coordinated the work between the two sites (UK & Egypt), recruited patients, obtained blood samples, performed AMH assays and collected data for analysis. AA and ARE recruited patients and collected blood samples in Minia Assisted Conception Unit. MKE reviewed the design and critically reviewed the manuscript. RWS reviewed the design, recruited patients and critically reviewed the manuscript. All authors read and approved the final manuscript.
